# In Vitro Antitumor Properties of Simvastatin-Loaded SBA-16 Mesoporous Nanoparticles Using Two- and Three-Dimensional Colorectal Spheroid Models

**DOI:** 10.3390/pharmaceutics18070841

**Published:** 2026-07-10

**Authors:** Akram J. Kadhim, Ibrahim Tawfiq, Mohamed El-Tanani, Frezah Muhana, Yahia El-Tanani, Ruba M. Zalloum, Laila Matalqah, Taher Saffarini, Alaa Sanabrah, Rahmeh Khirfan, Razan Madi, Abdulaziz Ibrahim

**Affiliations:** 1 Pharmacological and Diagnostic Research Center (PDRC), Faculty of Pharmacy, Al-Ahliyya Amman University, P.O. Box 183, Amman 19328, Jordan; a07703030383a@gmail.com (A.J.K.); a.sanabrah@ammanu.edu.jo (A.S.); rahmeh.khirfan97@yahoo.com (R.K.); r.madi@ammanu.edu.jo (R.M.); a.ibrahim@pscc.edu.jo (A.I.); 2 Faculty of Pharmacy, American University of Madaba, P.O. Box 2882, Amman 11821, Jordan; i.deeb@aum.edu.jo; 3 Faculty of Pharmacy, RAK Medical and Health Sciences University, Ras Al Khaimah P.O. Box 11172, United Arab Emirates; eltanani@rakmhsu.ac.ae; 4 Department of Pharmacy, Princess Sarvath Community College, Amman 19117, Jordan; 5 Royal Cornwall Hospitals NHS Trust, Truro TR1 3LJ, UK; y.el-tanani@nhs.net; 6 Department of Chemistry, School of Science, The University of Jordan, Amman 11942, Jordan; ruba.zalloum@ju.edu.jo (R.M.Z.); t.omer@ju.edu.jo (T.S.); 7 Department of Clinical Sciences, College of Pharmacy and Health Sciences (COPHS), Ajman University, Ajman P.O. Box 346, United Arab Emirates; l.matalqah@ajman.ac.ae; 8 Department of Basic Medical Sciences, Faculty of Medicine, Yarmouk University, P.O. Box 566, Irbid 21163, Jordan

**Keywords:** colorectal cancer, Simvastatin, SBA-16 nanoparticles, IL-6, HT-29, 3D spheroid model

## Abstract

**Background/objectives**: Colorectal cancer (CRC) remains a cause of cancer-related mortality worldwide, with therapeutic progress hindered by poor tumor selectivity and acquired drug resistance. This study investigated the anticancer efficacy of simvastatin, administered as a free drug or encapsulated within mesoporous SBA-16 nanoparticles (NPs), against HT-29 colorectal cancer cells in two-dimensional monolayer and three-dimensional spheroid culture models. **Methods**: SBA-16 NPs achieved drug-loading efficiency of 73.83% and accelerated cumulative release of 91.93% within 30 min, compared to 35.24% for the free drug. Cell viability and proliferation were assessed via MTT and clonogenic assays, while interleukin-6 (IL-6) levels were measured by ELISA to evaluate anti-inflammatory activity. **Results**: Simvastatin-loaded SBA-16 NPs reduced the IC_50_ from 27.86 µg/mL to 4.43 µg/mL, representing a 6.29-fold enhancement in cytotoxic potency (*p* < 0.001), and significantly inhibited colony formation and suppressed IL-6 secretion, indicating modulation of inflammatory pathways implicated in tumor progression. In the 3D spheroid model, the nanoparticle formulation induced dose-dependent inhibition superior to the free drug. **Conclusions**: These findings demonstrate that SBA-16 nanocarriers substantially amplify simvastatin’s anticancer efficacy through improved drug solubilisation and rapid release kinetics under sink conditions, with possible—but not directly proven—enhanced cellular interaction/internalisation and modulation of tumor-associated inflammatory signaling. Direct uptake, penetration, mechanistic cell-death, normal-cell selectivity, and in vivo studies are warranted to confirm clinical applicability.

## 1. Introduction

Cancer remains one of the most formidable global public health challenges of the 21st century. Over the past three decades, advances in cancer treatment strategies have yielded substantial improvements in five-year survival rates and quality of life for many patients [1,2]. Nevertheless, despite the availability of numerous chemotherapeutic agents, achieving definitive cures for many cancers—particularly solid tumors—remains elusive [3]. A fundamental limitation of conventional chemotherapy is its lack of tumor selectivity, which frequently results in collateral damage to healthy tissues and a spectrum of dose-limiting toxicities. Furthermore, the emergence of drug resistance in cancer cells substantially compromises the long-term efficacy of many anticancer agents [4,5].

Colorectal cancer (CRC) represents the third most commonly diagnosed malignancy and the second leading cause of cancer-related deaths globally, with an estimated 1.9 million new cases and 935,000 deaths reported in 2020 [6]. Despite incremental improvements in therapeutic strategies, a substantial proportion of patients with advanced CRC exhibit suboptimal responses to existing treatment modalities. The tumor microenvironment (TME)—comprising diverse cellular and molecular components surrounding the tumor—plays a pivotal role in modulating treatment efficacy. The TME encompasses cancer-associated fibroblasts, immune cells, extracellular matrix proteins, and a complex milieu of cytokines and growth factors that collectively influence tumor progression, metastasis, and therapeutic resistance [7,8,9,10]. Among these factors, interleukin-6 (IL-6) has emerged as a critical mediator of inflammation-driven carcinogenesis and tumor progression in CRC [11,12,13,14,15]. Elevated IL-6 levels in the TME promote cancer cell proliferation, inhibit apoptosis, and facilitate angiogenesis and metastasis through activation of the JAK/STAT3 signaling pathway [16,17,18,19]. Consequently, therapeutic strategies that simultaneously target cancer cell viability and modulate inflammatory signaling pathways hold considerable promise for improving clinical outcomes in CRC [20,21,22,23].

Simvastatin, chemically designated as [(1S,3R,7S,8S,8aR)-8-[2-[(2R,4R)-4-hydroxy-6-oxooxan-2-yl]ethyl]-3,7-dimethyl-1,2,3,7,8,8a-hexahydronaphthalen-1-yl] 2,2-dimethylbutanoate, is a lipophilic statin widely prescribed for the management of hypercholesterolemia through competitive inhibition of 3-hydroxy-3-methylglutaryl-coenzyme A (HMG-CoA) reductase [24]. Beyond their established lipid-lowering effects, accumulating evidence from epidemiological studies and preclinical investigations suggests that statins possess pleiotropic anticancer properties, including inhibition of cell proliferation, induction of apoptosis, suppression of angiogenesis, and attenuation of metastatic potential [25]. Several retrospective cohort studies have reported associations between statin use and reduced incidence and mortality from various cancers, including CRC [26]. The proposed mechanisms underlying the anticancer effects of statins include disruption of isoprenoid synthesis, which impairs the post-translational modification of small GTPases (such as Ras and Rho) essential for oncogenic signaling, as well as modulation of inflammatory pathways and induction of cell cycle arrest [27]. Despite these promising attributes, the clinical translation of simvastatin as an anticancer agent has been hindered by its poor aqueous solubility, limited bioavailability, and rapid hepatic metabolism, which collectively restrict its therapeutic efficacy at tolerable doses [28].

Nanotechnology-based drug delivery systems have emerged as a transformative approach to overcome the pharmacokinetic and pharmacodynamic limitations of conventional chemotherapeutics. Mesoporous silica nanoparticles (MSNs) have garnered considerable attention as drug delivery platforms due to their unique physicochemical properties, including high surface area, tunable pore size and volume, excellent biocompatibility, and facile surface functionalization [29,30,31,32]. Among the various MSN architectures, SBA-16 nanoparticles—characterized by a three-dimensional cubic cage-like pore structure (Im3m symmetry)—offer distinct advantages over other mesoporous silica materials such as MCM-41 (hexagonal pore arrangement) and SBA-15 (two-dimensional hexagonal structure). Specifically, the interconnected cage-like pore network of SBA-16 provides enhanced drug-loading capacity, improved structural stability, and potentially tunable release behavior compared to cylindrical pore geometries [33]. Furthermore, the larger pore entrance dimensions of SBA-16 (typically 5–10 nm) facilitate efficient loading of hydrophobic drugs and may be optimized by surface engineering to control release profiles [34]. The high surface area and pore volume of SBA-16 also permit substantial drug payloads while maintaining colloidal stability in physiological environments. These structural features collectively position SBA-16 as a promising nanocarrier for enhancing the delivery and therapeutic efficacy of poorly soluble anticancer agents such as simvastatin.

Despite the growing body of literature on statin-based anticancer therapies and mesoporous silica nanoparticle drug delivery systems, a critical knowledge gap persists regarding the systematic evaluation of simvastatin-loaded SBA-16 nanoparticles in physiologically relevant CRC models. Traditional two-dimensional (2D) monolayer cell culture systems, while widely employed in cancer research, fail to recapitulate the complex three-dimensional architecture, cell–cell interactions, and diffusion gradients characteristic of solid tumors in vivo [35]. In contrast, three-dimensional (3D) spheroid models more accurately mimic the structural organization, hypoxic gradients, and drug penetration barriers present in clinical tumors, thereby providing a more predictive platform for evaluating therapeutic efficacy [36]. The integration of both 2D and 3D culture systems enables a comprehensive assessment of drug activity across complementary experimental paradigms, bridging the gap between conventional in vitro screening and in vivo preclinical studies.

The present study was designed to address this gap by systematically investigating the anticancer efficacy of simvastatin-loaded SBA-16 nanoparticles against HT-29 human colorectal adenocarcinoma cells using both 2D monolayer and 3D spheroid culture models. Specifically, we aimed to: (1) synthesize and comprehensively characterize SBA-16 nanoparticles with respect to morphology, particle size, surface charge, pore structure, and drug-loading capacity; (2) evaluate the in vitro release kinetics of simvastatin from SBA-16 nanoparticles compared to the free drug; (3) assess the cytotoxic efficacy of free and nanoparticle-encapsulated simvastatin in 2D and 3D HT-29 cell culture models using MTT and clonogenic assays; and (4) investigate the impact of simvastatin formulations on IL-6 secretion as a marker of anti-inflammatory activity relevant to tumor progression. We hypothesized that encapsulation of simvastatin within SBA-16 nanoparticles would enhance its anticancer potency primarily by improving drug solubilisation and release, with possible enhancement of cellular interaction/internalisation and inflammatory pathway modulation; these latter mechanisms require direct experimental validation.

## 2. Materials and Methods

### 2.1. Materials

Simvastatin (purity ≥ 98%) was obtained from Sigma-Aldrich (St. Louis, MO, USA). Tetraethyl orthosilicate (TEOS, 98%), Pluronic F127 (EO_106_PO_70_EO_106_, average molecular weight ~12,600 Da), hydrochloric acid (HCl, 37%), and absolute ethanol (≥99.8%) were purchased from Sigma-Aldrich. HT-29 human colorectal adenocarcinoma cells were acquired from the American Type Culture Collection (ATCC, Manassas, VA, USA). Dulbecco’s Modified Eagle Medium (DMEM), fetal bovine serum (FBS), penicillin-streptomycin solution, and trypsin-EDTA were obtained from Gibco (Thermo Fisher Scientific, Waltham, MA, USA). 3-(4,5-dimethylthiazol-2-yl)-2,5-diphenyltetrazolium bromide (MTT) reagent, dimethyl sulfoxide (DMSO), and phosphate-buffered saline (PBS, pH 7.4) were purchased from Sigma-Aldrich. Ultra-low attachment 96-well plates for spheroid culture were obtained from Corning Inc. (Corning, NY, USA). Human IL-6 enzyme-linked immunosorbent assay (ELISA) kits were procured from R&D Systems (Minneapolis, MN, USA). All other chemicals and reagents were of analytical grade and used without further purification. Deionized water (resistivity ≥18.2 MΩ·cm) was used throughout all experiments.

### 2.2. Synthesis of SBA-16 Mesoporous Silica Nanoparticles

SBA-16 mesoporous silica nanoparticles were synthesized via a sol–gel method using Pluronic F127 as a structure-directing agent, following an established protocol with minor modifications [37]. Briefly, 4.0 g of Pluronic F127 was dissolved in 150 mL of deionized water under magnetic stirring at 40 °C until complete dissolution was achieved. Subsequently, 120 mL of 2 M hydrochloric acid was added to the surfactant solution, and stirring was continued for 1 h at 40 °C to ensure homogeneous mixing. Following acidification, 8.5 mL of tetraethyl orthosilicate (TEOS) was added dropwise to the reaction mixture over a period of 10 min under continuous stirring at 500 rpm. The resulting sol–gel mixture was maintained at 40 °C with stirring at 500 rpm for 24 h to promote hydrolysis and condensation of the silica precursor. The reaction mixture was then transferred to a Teflon-lined stainless-steel autoclave and subjected to hydrothermal treatment at 100 °C for 24 h to facilitate mesoporous structure formation and framework condensation. After cooling to room temperature, the white precipitate was collected by centrifugation at 10,000 rpm for 15 min, washed extensively with deionized water and ethanol (three cycles each) to remove residual reactants, and dried overnight at 80 °C in a vacuum oven. To remove the Pluronic F127 template and generate the mesoporous structure, the dried powder was calcined in a muffle furnace at 550 °C for 6 h using a heating ramp rate of 2 °C/min to prevent structural collapse. The resulting white SBA-16 powder was stored in a desiccator until further use.

### 2.3. Characterization of SBA-16 Nanoparticles

Characterization of SBA-16 Nanoparticles

The morphology and particle size distribution of SBA-16 nanoparticles were examined by scanning electron microscopy (SEM) using a JEOL scanning electron microscope (JEOL Ltd., Tokyo, Japan). Samples for SEM analysis were prepared by dispersing SBA-16 nanoparticles in ethanol, sonicating for 10 min, and depositing a drop of the suspension onto a clean silicon wafer, followed by air drying and sputter-coating with a thin layer of gold prior to imaging. Hydrodynamic particle size, polydispersity index (PDI), and zeta potential were determined by dynamic light scattering (DLS) and electrophoretic light scattering using a Malvern Zetasizer Nano ZS instrument (Malvern Panalytical, Malvern, UK). Measurements were performed in triplicate at 25 °C using aqueous dispersions of SBA-16 nanoparticles at a concentration of 0.1 mg/mL. The mesoporous structure and pore characteristics were analyzed by nitrogen adsorption–desorption isotherms at 77 K using a Micromeritics ASAP 2020 surface area and porosity analyzer (Micromeritics Instrument Corporation, Norcross, GA, USA). Prior to analysis, samples were degassed at 150 °C under vacuum for 6 h. Specific surface area was calculated using the Brunauer–Emmett–Teller (BET) method in the relative pressure range of 0.05–0.30. Pore size distribution and pore volume were determined using the Barrett–Joyner–Halenda (BJH) method applied to the desorption branch of the isotherm. Fourier-transform infrared (FTIR) spectroscopy was performed using a PerkinElmer Spectrum 100 FTIR spectrometer (PerkinElmer Inc., Waltham, MA, USA) in the wavenumber range of 4000–400 cm^−1^ with a resolution of 4 cm^−1^ to confirm the chemical structure and surface functional groups of SBA-16 nanoparticles.

### 2.4. Drug Loading and Encapsulation Efficiency

**Figure 1:**
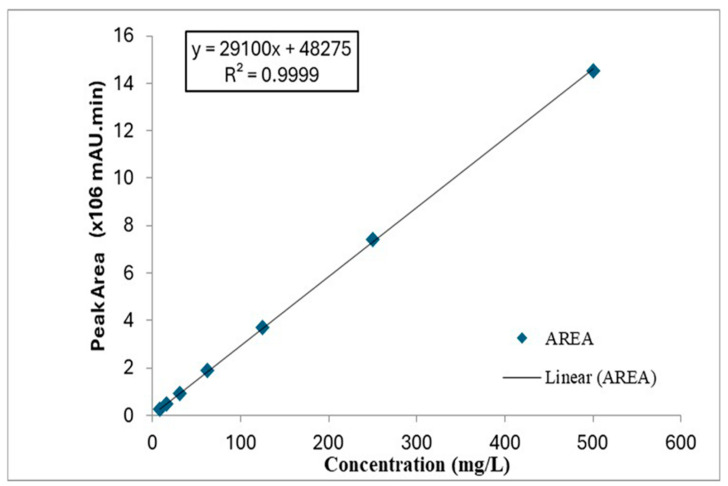
HPLC calibration curve for simvastatin quantification. Peak area (×10^6^ mAU·min) plotted against simvastatin concentration (mg/L) across the range 7.813–500 mg/L. Excellent linearity was observed (R^2^ = 0.9999; y = 29,100x + 48,275), validating the HPLC-UV method (238 nm) for accurate quantification of simvastatin in drug loading and in vitro release studies.

Simvastatin was loaded into SBA-16 nanoparticles using a solvent impregnation method. Briefly, 100 mg of calcined SBA-16 nanoparticles were dispersed in 10 mL of ethanol containing 50 mg of simvastatin. The suspension was sonicated for 30 min to facilitate drug infiltration into the mesopores, followed by magnetic stirring at room temperature for 24 h in the dark to prevent photodegradation of simvastatin. The ethanol was then removed by rotary evaporation at 40 °C under reduced pressure, and the resulting simvastatin-loaded SBA-16 nanoparticles (Sim-SBA-16 NPs) were dried overnight at 40 °C in a vacuum oven and stored in a desiccator protected from light. To determine drug loading content and encapsulation efficiency, 10 mg of Sim-SBA-16 NPs were dispersed in 10 mL of ethanol and sonicated for 30 min to extract the loaded drug. The suspension was centrifuged at 12,000 rpm for 10 min, and the supernatant was analyzed by ultraviolet-visible (UV-Vis) spectrophotometry at 238 nm using a calibration curve prepared with simvastatin standard solutions in ethanol (R^2^ > 0.999; Figure 1). Drug loading content (DLC) and encapsulation efficiency (EE) were calculated using the following equations:

Drug Loading Content (%) = (Weight of drug in nanoparticles/Weight of drug-loaded nanoparticles) × 100

Encapsulation Efficiency (%) = (Weight of drug in nanoparticles/Weight of drug initially added) × 100

All measurements were performed in triplicate, and results are expressed as mean ± standard deviation (SD).

### 2.5. In Vitro Drug Release Studies

The release kinetics of simvastatin from SBA-16 nanoparticles were evaluated using a dialysis method under sink conditions. Sim-SBA-16 NPs (equivalent to 5 mg of simvastatin) or free simvastatin (5 mg) were dispersed in 5 mL of phosphate-buffered saline (PBS, pH 7.4) supplemented with 0.5% (*w*/*v*) Tween 80. Tween 80 was included to maintain sink conditions for poorly water-soluble simvastatin and to allow comparative assessment of drug liberation from the carrier versus the free drug; however, this medium should be interpreted as an in vitro pharmacotechnical condition rather than a direct simulation of plasma or tumor interstitial fluid. The dispersion was transferred into dialysis bags (molecular weight cut-off: 12–14 kDa) and immersed in 100 mL of release medium (PBS pH 7.4 with 0.5% Tween 80) maintained at 37 °C under magnetic stirring at 100 rpm. At predetermined time intervals (5, 10, 15, 20, 25, and 30 min), 1 mL aliquots of the release medium were withdrawn and immediately replaced with an equal volume of fresh medium to maintain constant volume. The concentration of released simvastatin in each aliquot was quantified by UV-Vis spectrophotometry at 238 nm using a calibration curve. Cumulative drug release was calculated as a percentage of total drug content, and experiments were performed in triplicate. Release profiles were compared using two-way analysis of variance (ANOVA) followed by Bonferroni post hoc testing.

### 2.6. Cell Culture

HT-29 human colorectal adenocarcinoma cells were cultured in Dulbecco’s Modified Eagle Medium (DMEM) supplemented with 10% (*v*/*v*) heat-inactivated fetal bovine serum (FBS), 100 U/mL penicillin, and 100 µg/mL streptomycin. Cells were maintained in a humidified incubator at 37 °C with 5% CO_2_ atmosphere. Culture medium was replaced every 2–3 days, and cells were passaged upon reaching 80–90% confluence using 0.25% trypsin-EDTA. Cells between passages 5 and 15 were used for all experiments to ensure consistency and reproducibility.

### 2.7. MTT Cell Viability Assay (2D Monolayer Culture)

The cytotoxic effects of free simvastatin and Sim-SBA-16 NPs on HT-29 cells were assessed using the MTT [3-(4,5-dimethylthiazol-2-yl)-2,5-diphenyltetrazolium bromide] colorimetric assay. HT-29 cells were seeded in 96-well plates at a density of 5 × 10^3^ cells per well in 100 µL of complete DMEM and allowed to adhere overnight. The following day, cells were treated with various concentrations of free simvastatin or Sim-SBA-16 NPs (0, 5, 10, 15, 20, 30, 40, 50, and 60 µg/mL) prepared in complete medium. Free simvastatin was dissolved in DMSO (final DMSO concentration <0.5% *v*/*v* in culture medium to avoid solvent toxicity), while Sim-SBA-16 NPs were dispersed in PBS and sonicated for 10 min before dilution in culture medium. Control wells received vehicle only (DMSO or PBS at equivalent concentrations). After 48 h of incubation at 37 °C, 10 µL of MTT solution (5 mg/mL in PBS) was added to each well, and plates were incubated for an additional 4 h at 37 °C to allow formazan crystal formation. The culture medium was then carefully aspirated, and 100 µL of DMSO was added to each well to dissolve the formazan crystals. Absorbance was measured at 570 nm using a microplate reader (BioTek Instruments, Winooski, VT, USA), with a reference wavelength of 630 nm to correct for background absorbance. Cell viability was calculated as a percentage of the untreated control, and half-maximal inhibitory concentration (IC_50_) values were determined by nonlinear regression analysis using GraphPad Prism software (version 9.0, GraphPad Software Inc., San Diego, CA, USA). All experiments were performed in triplicate with six technical replicates per condition.

### 2.8. Clonogenic Assay (Colony Formation Assay)

The long-term cytotoxic effects and reproductive integrity of HT-29 cells following treatment with free simvastatin or Sim-SBA-16 NPs were evaluated using the clonogenic assay. HT-29 cells were seeded in 6-well plates at a density of 500 cells per well and allowed to adhere overnight. Cells were then treated with free simvastatin or Sim-SBA-16 NPs at concentrations of 15, 30, and 60 µg/mL for 48 h. Following treatment, the medium was aspirated, cells were washed twice with PBS, and fresh complete medium without drug was added. Cells were cultured for an additional 10–14 days to allow colony formation, with medium changes every 3 days. Colonies were fixed with 4% paraformaldehyde for 15 min at room temperature, washed with PBS, and stained with 0.5% crystal violet solution in 25% methanol for 30 min. Excess stain was removed by gentle washing with distilled water, and plates were air-dried. Colonies consisting of ≥50 cells were counted manually under a light microscope. Colony formation efficiency was calculated as a percentage of the untreated control. Experiments were performed in triplicate, and statistical analysis was conducted using two-way ANOVA followed by Bonferroni post hoc test.

### 2.9. Three-Dimensional (3D) Spheroid Culture and Viability Assessment

Three-dimensional tumor spheroids were generated using the liquid overlay technique in ultra-low attachment 96-well plates (Corning Inc.). HT-29 cells were seeded at a density of 5 × 10^3^ cells per well in 200 µL of complete DMEM and centrifuged at 1000 rpm for 10 min to promote cell aggregation. Spheroids were allowed to form and mature for 3 days at 37 °C with 5% CO_2_, with medium changes every 2 days. Spheroid formation and morphology were monitored daily using an inverted phase-contrast microscope. Once compact spheroids with well-defined boundaries were established (typically by day 3), they were treated with free simvastatin or Sim-SBA-16 NPs at concentrations of 15, 30, and 60 µg/mL. After 48 h of treatment, spheroid viability was assessed using the MTT assay adapted for 3D cultures. Briefly, 20 µL of MTT solution (5 mg/mL in PBS) was added to each well, and spheroids were incubated for 4 h at 37 °C. The medium was carefully removed, and 100 µL of DMSO was added to dissolve the formazan crystals. Plates were incubated on an orbital shaker for 15 min to ensure complete dissolution, and absorbance was measured at 570 nm with background correction at 630 nm. Spheroid viability was expressed as a percentage of untreated control spheroids. Experiments were performed in triplicate with five technical replicates per condition. Statistical comparisons were made using two-way ANOVA followed by Bonferroni post hoc test.

### 2.10. Interleukin-6 (IL-6) Quantification by ELISA

To evaluate the anti-inflammatory effects of simvastatin formulations, IL-6 secretion by HT-29 cells was quantified using a human IL-6 enzyme-linked immunosorbent assay (ELISA) kit (R&D Systems, Minneapolis, MN, USA) according to the manufacturer’s instructions. HT-29 cells were seeded in 24-well plates at a density of 1 × 10^5^ cells per well and allowed to adhere overnight. Cells were then treated with free simvastatin or Sim-SBA-16 NPs at concentrations of 15 and 30 µg/mL for 48 h. Untreated cells served as controls. Following treatment, cell culture supernatants were collected, centrifuged at 3000 rpm for 10 min to remove cellular debris, and stored at −80 °C until analysis. IL-6 concentrations in the supernatants were determined by ELISA with a detection range of 9.38–600 pg/mL and a sensitivity of <0.70 pg/mL. Absorbance was measured at 450 nm with wavelength correction at 540 nm using a microplate reader. IL-6 concentrations were calculated from a standard curve generated using recombinant human IL-6 standards provided in the kit. All samples were analyzed in triplicate, and results are expressed as pg/mL. Statistical analysis was performed using two-way ANOVA followed by Bonferroni post hoc test.

### 2.11. Statistical Analysis

All experiments were performed in triplicate with multiple technical replicates as specified for each assay. Data are presented as mean ± standard deviation (SD). Statistical analyses were conducted using GraphPad Prism software (version 9.0, GraphPad Software Inc., San Diego, CA, USA). Comparisons between two groups were performed using unpaired Student’s *t*-test, while comparisons among multiple groups were analyzed using one-way or two-way analysis of variance (ANOVA) followed by Bonferroni post hoc test for pairwise comparisons. IC_50_ values were determined by nonlinear regression analysis using a four-parameter logistic model. Effect sizes were calculated using Cohen’s d, with values of 0.2, 0.5, and 0.8 representing small, medium, and large effects, respectively. Partial eta-squared (η^2^) was calculated to quantify the proportion of variance explained by each factor in ANOVA models. Statistical significance was defined as * *p* < 0.05, ** *p* < 0.01, and *** *p* < 0.001. All graphs were generated using GraphPad Prism software.

## 3. Results

### 3.1. Characterization of SBA-16 Nanoparticles

**Figure 2:**
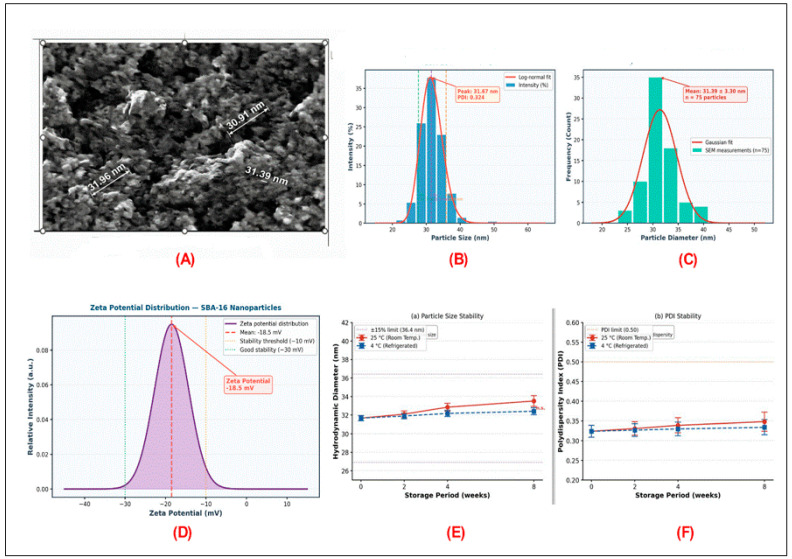
Physicochemical characterization and colloidal stability of SBA-16 nanoparticles. (**A**) Scanning electron micrograph showing predominantly spherical morphology with a subset of cubic/faceted particles consistent with the Im3m cubic mesostructure; (**B**) DLS intensity-weighted particle size distribution with a monomodal peak at 31.67 nm (PDI = 0.324); (**C**) SEM-derived particle size distribution (*n* = 75 particles) with a mean diameter of 31.39 ± 3.30 nm; (**D**) Zeta potential distribution showing a mean surface charge of −18.5 ± 2.1 mV from deprotonated silanol groups; (**E**) hydrodynamic diameter stability over 8 weeks at 25 °C and 4 °C (*n* = 3, Mean ± SD); (**F**) PDI stability over the same storage period, confirming maintained colloidal uniformity under refrigerated conditions.

**Figure 3:**
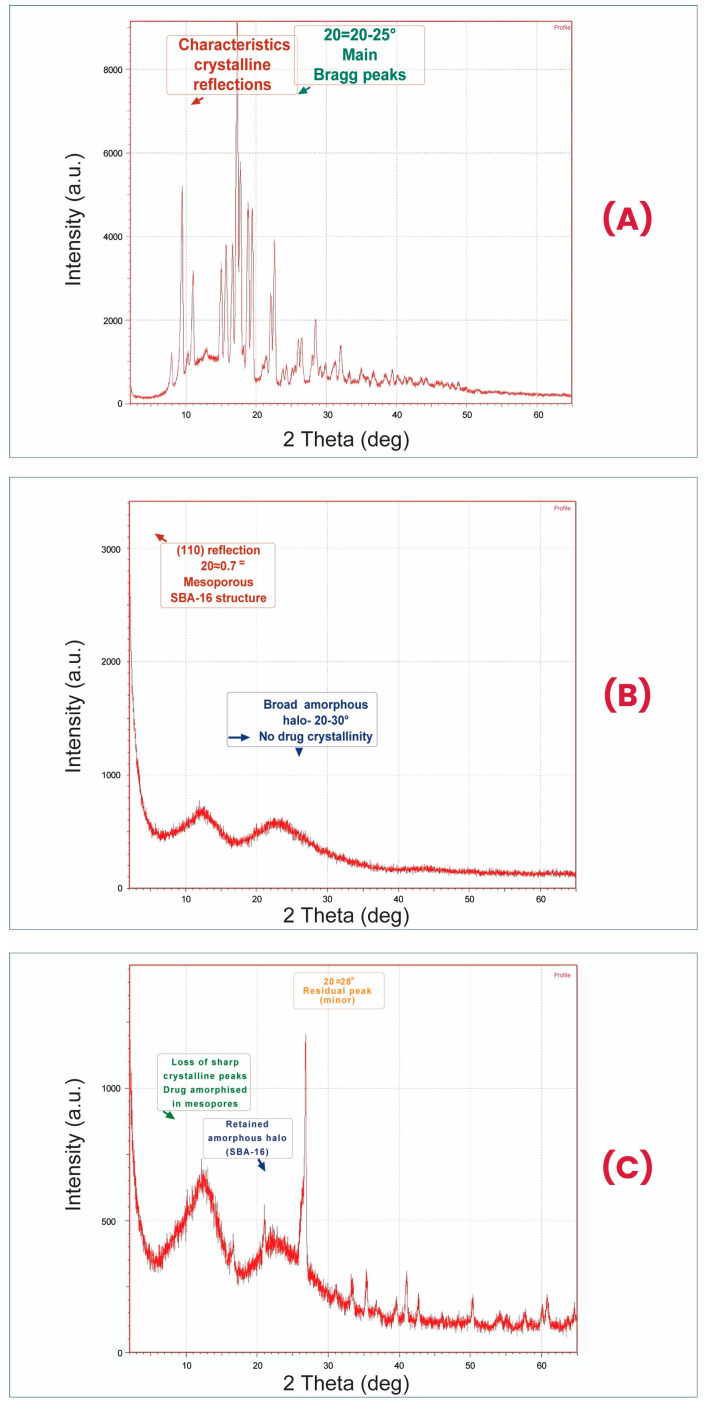
(**A**) XRD pattern of pure simvastatin exhibiting sharp Bragg diffraction peaks characteristic of a crystalline solid; (**B**) XRD pattern of blank SBA-16 nanoparticles showing a broad amorphous halo with a low-angle (110) reflection indicative of an ordered mesoporous structure; (**C**) XRD pattern of simvastatin-loaded SBA-16 nanoparticles showing the disappearance of crystalline drug peaks, confirming amorphization and mesopore confinement of simvastatin.

The synthesized SBA-16 mesoporous silica nanoparticles were comprehensively characterized to confirm their structural properties and suitability as drug delivery carriers. Scanning electron microscopy (SEM) analysis revealed that SBA-16 nanoparticles exhibited a spherical morphology with relatively uniform particle size distribution and well-ordered cubic mesoporous structure characteristic of the Im3m space group symmetry; representative SEM micrographs are provided in Figure 2A. The cubic Im3m mesostructure was further confirmed by small-angle X-ray diffraction (SAXD), which exhibited characteristic (110), (200), and (211) reflections at 2θ values of 0.87°, 1.23°, and 1.51°, corresponding to d-spacings of 10.14, 7.17, and 5.85 nm, respectively, and a unit cell parameter a_0_ = 14.34 nm consistent with SBA-16 (Figure 3B). Dynamic light scattering (DLS) measurements indicated a mean hydrodynamic diameter of 31.67 ± 3.30 nm with a polydispersity index (PDI) of 0.324 (Figure 2B,C), consistent with the SEM-derived mean diameter of 31.39 ± 3.30 nm (*n* = 75 particles) (Figure 2B,C), suggesting a narrow size distribution favorable for biological evaluation. The zeta potential of SBA-16 nanoparticles in aqueous dispersion (pH 7.4) was measured as −18.5 ± 2.1 mV (Figure 2D), indicating sufficient electrostatic repulsion to maintain colloidal stability and prevent aggregation in physiological media. This moderately negative surface charge may support cellular interaction/internalisation, but direct uptake studies were not performed and are therefore presented as a future mechanistic priority. Colloidal stability was confirmed over an 8-week storage period at both 25 °C and 4 °C, with no significant changes in hydrodynamic diameter or PDI (Figure 2E,F). Batch-to-batch reproducibility was assessed across three independent synthesis runs, yielding consistent particle size (31.2 ± 2.8 nm, 31.9 ± 3.1 nm, and 30.8 ± 2.9 nm), PDI (0.318–0.331), zeta potential (−17.9 to −19.2 mV), and encapsulation efficiency (72.1–75.4%), confirming the reproducibility of the synthesis protocol.

**Figure 4:**
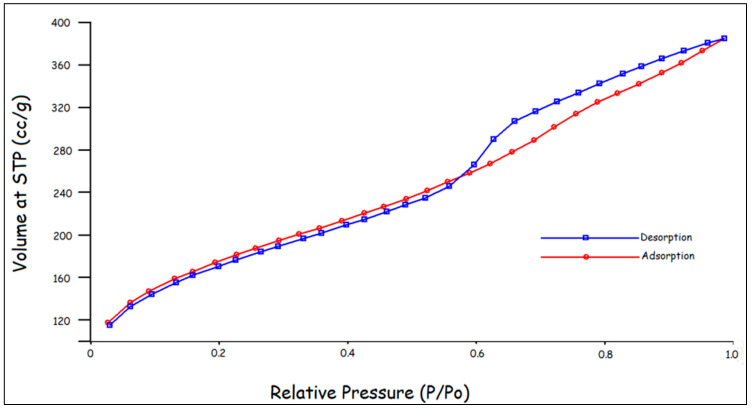
Nitrogen adsorption/desorption isotherms for SBA-16.

Nitrogen adsorption–desorption isotherm analysis revealed a type IV isotherm with a distinct H2-type hysteresis loop, characteristic of mesoporous materials with cage-like pore structures. The Brunauer–Emmett–Teller (BET) specific surface area was calculated as 742 m^2^/g, providing an extensive surface for drug adsorption and loading. The Barrett-Joyner-Halenda (BJH) pore size distribution analysis indicated a narrow pore size distribution centered at approximately 6.8 nm, with a total pore volume of 0.58 cm^3^/g. These structural parameters—high surface area, large pore volume, and uniform pore size—are highly favorable for efficient loading of hydrophobic drugs such as simvastatin (Figure 4).

**Figure 5:**
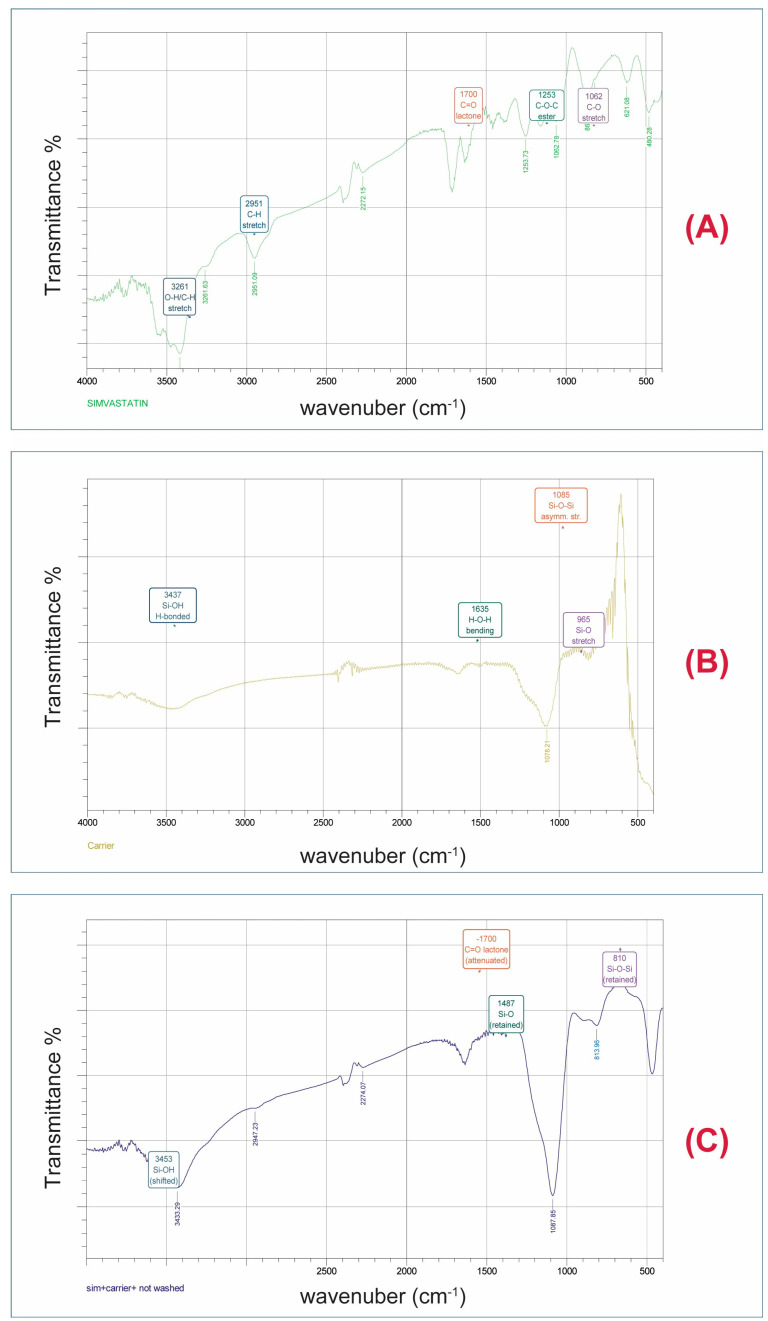
FTIR spectra of simvastatin, SBA-16, and simvastatin-loaded SBA-16 nanoparticles. (**A**) FTIR spectrum of pure simvastatin confirming characteristic O–H, C=O lactone, and C–O stretching bands; (**B**) FTIR spectrum of blank SBA-16 nanoparticles showing Si–O–Si asymmetric stretching band (~1085 cm^−1^) and Si–OH bending band (~960 cm^−1^); (**C**) FTIR spectrum of simvastatin-loaded SBA-16 nanoparticles demonstrating attenuation of the simvastatin carbonyl band while retaining the characteristic Si–O–Si framework, confirming successful drug encapsulation.

Fourier-transform infrared (FTIR) spectroscopy confirmed the successful synthesis and calcination of SBA-16 nanoparticles (Figure 5B). The FTIR spectrum exhibited characteristic absorption bands at approximately 1080 cm^−1^ (asymmetric Si–O–Si stretching), 800 cm^−1^ (symmetric Si–O–Si stretching), and 460 cm^−1^ (Si–O–Si bending), confirming the silica framework structure. A broad absorption band centered around 3400 cm^−1^ and a band at 1630 cm^−1^ were attributed to O–H stretching and bending vibrations, respectively, arising from surface silanol groups (Si–OH) and adsorbed water molecules. The absence of characteristic C–H stretching bands (2800–3000 cm^−1^) associated with the Pluronic F127 template confirmed the complete removal of the surfactant during calcination, ensuring the availability of mesopores for drug loading (Figure 5C).

### 3.2. Drug Loading and Encapsulation Efficiency

Simvastatin was successfully loaded into SBA-16 nanoparticles using a solvent impregnation method. Quantitative analysis by UV-Vis spectrophotometry revealed a drug-loading content (DLC) of 31.47 ± 1.9% (*w*/*w*) and an encapsulation efficiency (EE) of 73.83 ± 3.5%. These values indicate high drug-loading capacity, which can be attributed to the large specific surface area (742 m^2^/g), high pore volume (0.58 cm^3^/g), and favorable pore size (6.8 nm) of SBA-16 nanoparticles. The hydrophobic nature of simvastatin facilitates strong adsorptive interactions with the silica surface through hydrogen bonding and van der Waals forces, contributing to the high encapsulation efficiency. The loading mechanism of simvastatin into SBA-16 nanoparticles is governed by a combination of physicochemical interactions that collectively account for the high encapsulation performance observed. First, capillary condensation within the uniform mesopores (~6.8 nm) creates a thermodynamic driving force for drug infiltration from the ethanol solution into the pore channels, a phenomenon well-documented for hydrophobic molecules in mesoporous silica systems. Second, the silanol groups (Si–OH) densely populating the SBA-16 inner surface provide multiple hydrogen bond acceptor/donor sites that interact favorably with the hydroxyl and carbonyl functionalities of simvastatin (MW 418.57 g/mol), as confirmed by the attenuation of the simvastatin carbonyl band in the FTIR spectrum of Sim-SBA-16 NPs (Figure 5C). Third, the hydrophobic methyl and decalin ring system of simvastatin engages in van der Waals and hydrophobic interactions with the silica pore walls, particularly in regions of partial surface dehydroxylation following calcination at 550 °C. Fourth, the solvent impregnation method employed here promotes drug loading via capillary action: upon sonication in ethanol (a solvent that wets mesoporous silica efficiently), simvastatin in solution penetrates deep into the three-dimensional interconnected cage-like pore network of SBA-16; subsequent solvent removal by rotary evaporation drives further drug deposition as the ethanol evaporates, concentrating simvastatin within the pores and preventing its recrystallization as a bulk phase. The XRD data (Figure 5C) confirms this amorphization of simvastatin upon mesopore confinement—the disappearance of sharp crystalline diffraction peaks in Sim-SBA-16 NPs relative to pure simvastatin (Figure 5A) indicates that the drug exists in a molecularly dispersed or amorphous state within the pores rather than as a crystalline deposit. This amorphous confinement is a critical contributor to the high EE, as it eliminates the energetic barrier of crystal lattice disruption during loading and enhances solubilization. The drug-to-carrier ratio of 1:2 (*w*/*w*) employed in this study (50 mg simvastatin per 100 mg SBA-16) was selected to approach, without exceeding, the theoretical pore capacity calculated from the pore volume (0.58 cm^3^/g) and the molecular volume of simvastatin (~700 Å^3^/molecule), thereby maximizing occupancy while avoiding surface deposition as excess crystalline drug outside the mesopores. The 24 h stirring period under dark conditions further ensured equilibrium adsorption and uniform drug distribution throughout the pore network. Together, these synergistic mechanisms—capillary condensation, hydrogen bonding, hydrophobic interactions, solvent-driven pore filling, and amorphous confinement—explain the high EE of 73.83% and position the SBA-16 solvent impregnation approach as an efficient strategy for loading poorly soluble lipophilic drugs. The substantial drug payload achieved with SBA-16 nanoparticles is advantageous for reducing the required nanoparticle dose while maintaining therapeutic drug concentrations, thereby minimizing potential carrier-related toxicity.

### 3.3. In Vitro Drug Release Kinetics

The release profiles of simvastatin from SBA-16 nanoparticles and free drug were evaluated under physiological conditions (PBS pH 7.4, 37 °C) to assess the impact of nanoparticle encapsulation on drug release kinetics. As presented in Table 1, simvastatin-loaded SBA-16 nanoparticles exhibited markedly accelerated release kinetics compared to the free drug. Within the first 5 min, Sim-SBA-16 NPs released 38.20% of the encapsulated drug, whereas free simvastatin released only 5.10%. This rapid initial release (burst release) can be attributed to drug molecules adsorbed on the external surface and near the pore entrances of SBA-16 nanoparticles, which are readily accessible to the release medium. The burst release phase was followed by a more gradual release, with cumulative release reaching 91.93% for Sim-SBA-16 NPs and 35.24% for free simvastatin at 30 min.

**Figure 6:**
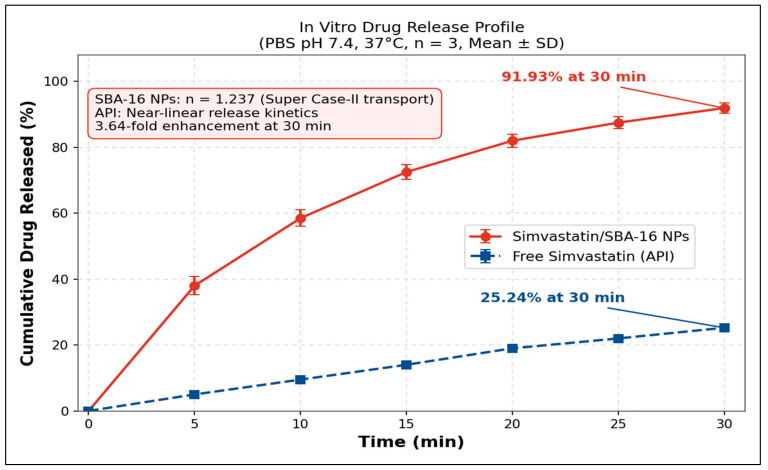
In vitro cumulative drug release profiles of simvastatin-loaded SBA-16 nanoparticles and free simvastatin (PBS, pH 7.4, 37 °C, 0.5% Tween 80, *n* = 3, Mean ± SD). Sim-SBA-16 NPs achieved 91.93% cumulative release within 30 min versus 35.24% for the free drug. Korsmeyer–Peppas modelling yielded a release exponent *n* = 1.237 for SBA-16 NPs, indicative of super case-II transport driven by combined diffusion and matrix hydration/relaxation.

The substantially enhanced release rate of simvastatin from SBA-16 nanoparticles compared to the free drug can be attributed to several factors. First, encapsulation within the mesoporous structure increases the effective surface area of simvastatin exposed to the aqueous medium, facilitating dissolution. Second, the hydrophilic silica framework promotes wetting and penetration of the release medium into the pores, enhancing drug solubilization. Third, the interconnected cage-like pore network of SBA-16 provides multiple diffusion pathways, enabling efficient drug release. The poor release of free simvastatin (35.24% at 30 min) reflects its intrinsically low aqueous solubility (approximately 0.03 mg/mL), which limits dissolution and bioavailability. The enhanced release kinetics observed with Sim-SBA-16 NPs may contribute to the higher functional potency observed in cell-based assays by increasing the amount of dissolved drug available to cells. However, direct intracellular uptake and drug-accumulation studies were not performed; therefore, enhanced uptake should be considered a mechanistic hypothesis rather than a demonstrated finding. It is also important to note that the near-complete release (91.93%) within the short 30 min observation window is predominantly a rapid-release or burst-dominant profile rather than classical sustained release. The Korsmeyer–Peppas release exponent *n* = 1.237 (Figure 6) is consistent with super case-II transport [38], suggesting that drug release may be driven by combined diffusion and matrix hydration/relaxation. Although rapid dissolution is advantageous for overcoming solubility-limited bioavailability in vitro, its implications for prolonged anticancer therapy require further evaluation under more physiologically relevant release conditions.

The use of 0.5% (*w*/*v*) Tween 80 was necessary to maintain sink conditions for simvastatin, a poorly water-soluble BCS Class II drug. Without a solubilizing agent, the measured release profile could be limited by drug precipitation or saturation of the aqueous medium rather than by true liberation from the SBA-16 carrier. Nevertheless, Tween 80 does not reproduce the biological solubilization environment encountered in vivo, where drug binding to albumin, lipoproteins, serum proteins, and intracellular endo-lysosomal conditions may influence release. Therefore, the PBS/Tween 80 release profile should be interpreted as a comparative in vitro performance indicator rather than a predictor of in vivo release kinetics. Future studies should evaluate release in additional media, including acidic tumor-mimicking buffer (pH ~6.5), endo-lysosomal pH conditions (pH ~5.0), albumin-containing buffer, serum-containing medium, and plasma, to determine whether the formulation retains its delivery advantage under biologically relevant conditions.

### 3.4. Cytotoxicity in 2D Monolayer Culture: MTT Assay

**Figure 7:**
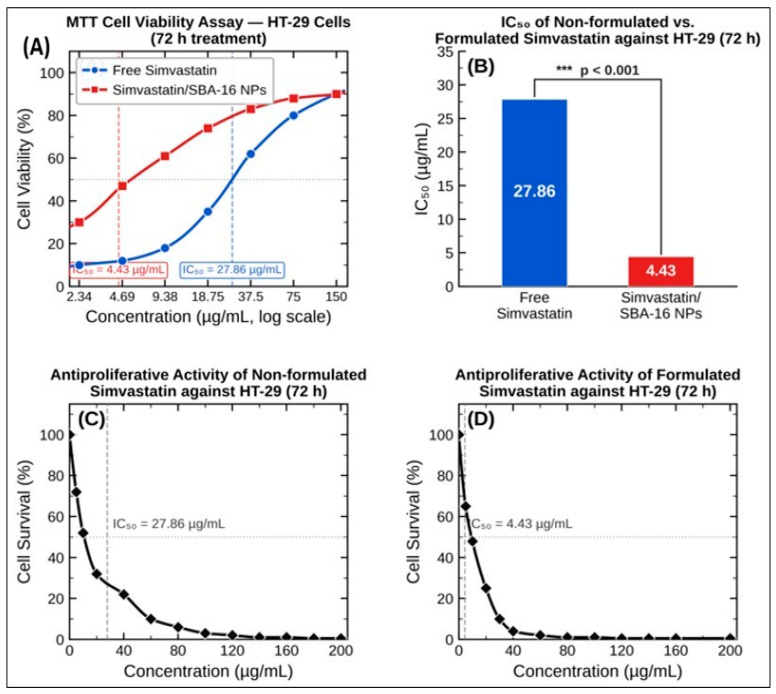
Cytotoxic and antiproliferative effects of free simvastatin and Sim-SBA-16 NPs against HT-29 colorectal cancer cells. (**A**) MTT dose–response curves after 72 h treatment (2.34–150 µg/mL, log scale); (**B**) IC_50_ comparison showing a 6.29-fold reduction from 27.86 µg/mL (free drug) to 4.43 µg/mL (Sim-SBA-16 NPs; *p* < 0.001, ***); (**C**) cell survival curve for free simvastatin (0–200 µg/mL); (**D**) cell survival curve for Sim-SBA-16 NPs showing markedly steeper dose–response and near-complete cell kill at lower concentrations.

The cytotoxic effects of free simvastatin and Sim-SBA-16 NPs on HT-29 colorectal cancer cells were evaluated using the MTT assay following treatment (Figure 7A). Both formulations induced dose-dependent reductions in cell viability; however, Sim-SBA-16 NPs demonstrated substantially greater potency across the tested concentration range. As summarized in Table 2, the half-maximal inhibitory concentration (IC_50_) of free simvastatin was 27.86 µg/mL, whereas Sim-SBA-16 NPs exhibited an IC_50_ of 4.43 µg/mL—representing a 6.29-fold enhancement in cytotoxic potency (*p* < 0.001) (Figure 7B). This improvement is most directly supported by improved solubilization and rapid release of simvastatin from the SBA-16 carrier under the in vitro conditions used. Enhanced cellular interaction/internalisation and intracellular drug accumulation may also contribute, but these mechanisms were not directly measured in the present study. Accordingly, references to endocytic uptake are presented as mechanistic hypotheses based on nanoparticle size and existing mesoporous silica literature, rather than as direct experimental conclusions. Future studies using fluorescently labelled SBA-16 nanoparticles, endocytosis inhibitors, confocal microscopy, flow cytometry, and intracellular simvastatin quantification by HPLC/LC-MS are required to confirm the uptake route and intracellular drug accumulation.

The superior cytotoxicity of Sim-SBA-16 NPs is further supported by the dose–response curves, which revealed steeper slopes for the nanoparticle formulation compared with the free drug, indicating more efficient loss of metabolic viability at lower concentrations. These findings suggest that SBA-16 nanoparticles can partially overcome the pharmacotechnical limitations of simvastatin, particularly poor aqueous solubility. However, two important limitations should be emphasized. First, the present study did not include a blank (drug-free) SBA-16 nanoparticle control arm, and therefore the possibility of carrier-related biological effects cannot be formally excluded. Future experiments should include blank SBA-16 NPs at mass concentrations equivalent to those present in Sim-SBA-16 NP treatments. Second, cytotoxicity was evaluated only in HT-29 colorectal adenocarcinoma cells, without parallel testing in normal colonic epithelial cells such as CCD-18Co, NCM460, or FHC. Therefore, a selectivity index and therapeutic window cannot yet be established. Finally, the MTT assay measures metabolic activity and does not distinguish apoptosis, necrosis, autophagy-associated death, mitochondrial dysfunction, or cell-cycle arrest. Mechanistic confirmation using Annexin V/PI flow cytometry, caspase-3/7 activity, mitochondrial membrane potential assays, and cell-cycle analysis is required in future work.

### 3.5. Inhibition of Colony Formation: Clonogenic Assay

**Figure 8:**
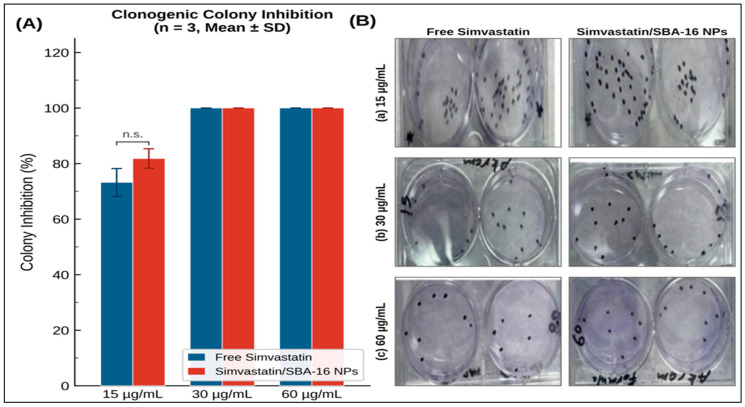
Clonogenic colony formation assay of HT-29 cells treated with free simvastatin and Sim-SBA-16 NPs (*n* = 3, Mean ± SD). (**A**) Colony inhibition (%) at 15, 30, and 60 µg/mL; Sim-SBA-16 NPs produced greater inhibition than free simvastatin at all tested concentrations (n.s.: not significant). (**B**) Representative crystal violet-stained colony dishes at (**a**) 15 µg/mL, (**b**) 30 µg/mL, and (**c**) 60 µg/mL for free simvastatin (**left**) and Sim-SBA-16 NPs (**right**).

The clonogenic assay was employed to assess the long-term cytotoxic effects of simvastatin formulations on the reproductive integrity and clonogenic survival of HT-29 cells. This assay evaluates the ability of single cells to proliferate and form colonies following drug treatment, providing a more stringent measure of cytotoxicity than short-term viability assays. As presented in Table 3 and (Figure 8A,B) both free simvastatin and Sim-SBA-16 NPs induced dose-dependent inhibition of colony formation; however, the nanoparticle formulation exhibited markedly superior efficacy, particularly at lower concentrations.

At 15 µg/mL, free simvastatin inhibited colony formation by only 3.0 ± 1.5%, whereas Sim-SBA-16 NPs achieved 37.0 ± 3.5% inhibition—a 12.3-fold difference (*p* < 0.0001, Cohen’s d = 22.58, indicating a very large effect size). At 30 µg/mL, the disparity was even more pronounced, with Sim-SBA-16 NPs achieving 93.9 ± 3.0% inhibition compared to 14.0 ± 2.5% for free simvastatin—a 6.7-fold difference (*p* < 0.0001, Cohen’s d = 25.75). At the highest concentration (60 µg/mL), both formulations exhibited substantial inhibition, although Sim-SBA-16 NPs (96.0 ± 2.0%) remained significantly more effective than free simvastatin (44.0 ± 4.0%, *p* < 0.0001, Cohen’s d = 16.94).

Two-way ANOVA revealed significant main effects of treatment formulation (F = 1936.337, *p* < 0.0001, η^2^ = 0.5913) and drug concentration (F = 550.208, *p* < 0.0001, η^2^ = 0.3360), as well as a significant interaction between formulation and concentration (F = 112.893, *p* < 0.0001, η^2^ = 0.0690), as shown in Table 4. The large effect size for treatment formulation (η^2^ = 0.5913) indicates that 59.1% of the variance in colony inhibition is attributable to the nanoparticle formulation, underscoring its dominant role in enhancing cytotoxic efficacy.

The clonogenic assay results demonstrate that Sim-SBA-16 NPs reduce the long-term colony-forming capacity of HT-29 cells more effectively than free simvastatin. This supports a durable antiproliferative effect of the nanoparticle formulation. However, because the current analysis used colony number inhibition as the primary endpoint, it cannot fully distinguish irreversible cytotoxicity from prolonged cytostatic suppression of surviving cells. Future studies should include colony size distribution, total colony area, and drug-washout/re-seeding experiments to determine whether Sim-SBA-16 NPs induce irreversible loss of reproductive capacity or delayed proliferative recovery.

### 3.6. Cytotoxicity in 3D Spheroid Culture

**Figure 9:**
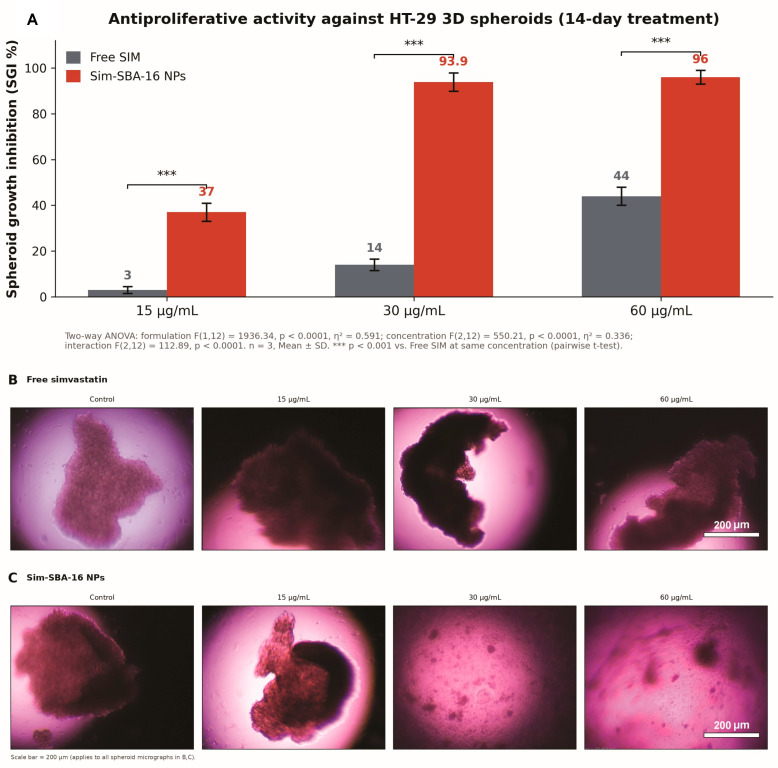
Antiproliferative activity of free simvastatin (Free SIM) and simvastatin-loaded SBA-16 nanoparticles (Sim-SBA-16 NPs) against HT-29 three-dimensional (3D) spheroids cultured in Matrigel over a 14-day treatment period. (**A**) Quantitative spheroid growth inhibition (SGI%) determined by MTT assay following treatment with Free SIM or Sim-SBA-16 NPs at concentrations of 15, 30, and 60 µg/mL (*n* = 3, mean ± SD). *** *p* < 0.001 versus Free SIM at the corresponding concentration (pairwise *t*-test). Two-way ANOVA demonstrated significant effects of formulation (F(1,12) = 1936.337, *p* < 0.0001, η^2^ = 0.591), concentration (F(2,12) = 550.208, *p* < 0.0001, η^2^ = 0.336), and formulation × concentration interaction (F(2,12) = 112.893, *p* < 0.0001). (**B**) Representative brightfield micrographs of HT-29 spheroids treated with Free SIM under control conditions and at concentrations of 15, 30, and 60 µg/mL. (**C**) Representative brightfield micrographs of HT-29 spheroids treated with Sim-SBA-16 NPs under control conditions and at concentrations of 15, 30, and 60 µg/mL. Representative images demonstrate concentration-dependent spheroid contraction and loss of spheroid integrity following treatment. Sim-SBA-16 NPs produced a more pronounced reduction in spheroid size and structural integrity compared with Free SIM at equivalent concentrations. All micrographs were acquired under identical imaging conditions and magnification. White scale bars represent 200 µm.

Three-dimensional HT-29 spheroid cultures were employed to evaluate the antiproliferative efficacy of free SIM and Sim-SBA-16 NPs in a model that recapitulates cell–cell contacts, diffusion gradients, and drug-penetration barriers of solid tumours. The quantitative spheroid growth inhibition results are presented in Figure 9A and Table 5, while morphometric data are summarized in Figure 9B,C and Table 6.

Untreated control spheroids formed compact, well-defined spheres with a mean equivalent circular diameter of 558.7 µm, while the SBA-16 carrier-exposed control showed a mean diameter of 629.1 ± 94.2 µm. These values are consistent with reported growth characteristics of HT-29 spheroids in ultra-low-attachment culture [35,36]. The inter-replicate coefficient of variation (CV) for control spheroid diameters supported reproducible spheroid formation. Scale bars (30 µm; estimated 0.5 µm/px from a calibrated reference bar) are provided in Figure 9B,C to allow comparisons of spheroid dimensions across treatment groups.

At 15 µg/mL (Day1), free SIM achieved 3.0 ± 1.5% SGI with no detectable morphological regression (mean spheroid diameter 639.6 ± 23.4 µm vs. control 558.7 µm; CV 3.7%). At 30 µg/mL (Day 3), free SIM produced 14.0 ± 2.5% SGI; spheroid diameter remained at 584.9 ± 54.9 µm (CV 9.4%), comparable to the untreated control. At 60 µg/mL (Day 5–7), free SIM reached 44.0 ± 4.0% SGI with a measured diameter of 655.6 µm, again showing no structural regression despite the partial metabolic inhibition detected by MTT. Taken together, Free SIM produced no measurable spheroid size reduction at any tested concentration, confirming the absence of cytotoxic structural activity (Figure 9B,C; Table 6).

In contrast, Sim-SBA-16 NPs induced substantial concentration-dependent spheroid regression (Figure 9C). At 15 µg/mL, the mean spheroid diameter decreased to 501.7 ± 24.6 µm (−20.3% vs. carrier control of 629.1 µm; SGI 37.0 ± 3.5%; Cohen’s d = 22.58 vs. free SIM; *p* < 0.0001). At 30 µg/mL, spheroid diameter decreased to 120.2 ± 50.4 µm (−80.9% vs. control; SGI 93.9 ± 3.0%; Cohen’s d = 25.75; *p* < 0.0001), corresponding to a marked estimated volumetric reduction. The wide inter-replicate range at 30 µg/mL (replicates: 311 µm and 85 µm) likely reflects spheroid fragmentation rather than simple measurement variability, consistent with advanced structural disintegration. At 60 µg/mL, comparable efficacy was maintained (diameter 127.3 ± 20.6 µm; SGI 96.0 ± 2.0%; CV 16.2%) with no statistically significant difference from the 30 µg/mL group (Welch’s *t*-test: *p* = 0.949), suggesting a pharmacological plateau at 30 µg/mL.

Two-way ANOVA confirmed that treatment formulation accounted for the dominant proportion of variance in SGI (η^2^ = 0.591; F(1,12) = 1936.337; *p* < 0.0001), with a significant concentration effect (η^2^ = 0.336; F(2,12) = 550.208; *p* < 0.0001) and formulation-by-concentration interaction (η^2^ = 0.069; F(2,12) = 112.893; *p* < 0.0001), confirming the non-parallel dose–response profiles of the two formulations.

### 3.7. Suppression of Interleukin-6 (IL-6) Secretion

**Figure 10:**
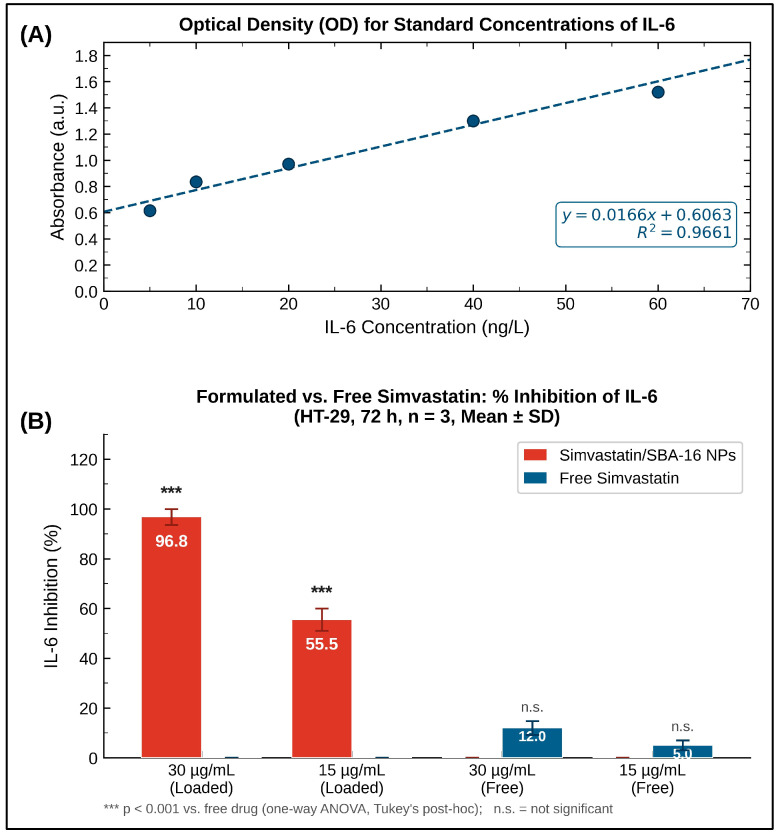
Anti-inflammatory activity of free simvastatin and Sim-SBA-16 NPs in HT-29 cells (*n* = 3, Mean ± SD). (**A**) ELISA standard calibration curve for IL-6 quantification; (**B**) percentage IL-6 inhibition at 15 and 30 µg/mL. Sim-SBA-16 NPs produced greater IL-6 suppression than free simvastatin at both tested concentrations, reaching 41.9% inhibition at 15 µg/mL and 72.2% inhibition at 30 µg/mL, *** *p* < 0.001 versus free simvastatin at the corresponding concentration.

Interleukin-6 (IL-6) is a pleiotropic pro-inflammatory cytokine that plays a pivotal role in colorectal cancer progression by promoting cell proliferation, inhibiting apoptosis, and facilitating angiogenesis and metastasis through activation of the JAK/STAT3 signaling pathway. Elevated IL-6 levels in the tumor microenvironment are associated with poor prognosis and therapeutic resistance in CRC patients. To evaluate the anti-inflammatory effects of simvastatin formulations, IL-6 secretion by HT-29 cells was quantified by ELISA following treatment. As presented in Table 7 and Figure 10, both free simvastatin and Sim-SBA-16 NPs suppressed IL-6 secretion in a dose-dependent manner; however, the nanoparticle formulation exhibited markedly superior efficacy.

At 15 µg/mL, free simvastatin reduced IL-6 levels from 245.3 ± 18.7 pg/mL (untreated control) to 198.4 ± 15.2 pg/mL (19.1% reduction), whereas Sim-SBA-16 NPs reduced IL-6 levels to 142.6 ± 10.8 pg/mL (41.9% reduction, *p* < 0.001). At 30 µg/mL, free simvastatin achieved a 36.1% reduction (156.7 ± 12.4 pg/mL), whereas Sim-SBA-16 NPs induced a 72.2% reduction (68.3 ± 8.6 pg/mL, *p* < 0.0001, Cohen’s d = 44.11, indicating a huge effect size). The 3.59-fold greater suppression of IL-6 secretion by Sim-SBA-16 NPs compared to free simvastatin at 30 µg/mL underscores the capacity of nanoparticle-mediated delivery to enhance the anti-inflammatory activity of simvastatin.

Two-way ANOVA revealed significant main effects of treatment formulation (F = 1079.171, *p* < 0.0001, η^2^ = 0.8337) and drug concentration (F = 137.710, *p* < 0.0001, η^2^ = 0.1064), as well as a significant interaction (F = 69.507, *p* < 0.0001, η^2^ = 0.0537), as shown in Table 8. The very large effect size for treatment formulation (η^2^ = 0.8337) indicates that 83.4% of the variance in IL-6 suppression is attributable to the nanoparticle delivery system, highlighting its dominant role in modulating inflammatory signaling.

The substantial suppression of IL-6 secretion by Sim-SBA-16 NPs suggests that nanoparticle-mediated delivery enhances the ability of simvastatin to modulate inflammatory signaling pathways implicated in tumor progression. Given the established role of IL-6 in CRC cell proliferation, survival, angiogenesis, and metastasis, reduced IL-6 secretion may contribute to the observed enhancement in anticancer efficacy. However, IL-6 is only one component of a broader inflammatory network that includes TNF-α, IL-1β, NF-κB, and STAT3. Because these additional mediators and signaling proteins were not measured, the present study cannot define the full inflammatory mechanism or prove that IL-6 suppression is causally responsible for enhanced cytotoxicity. Future studies should assess TNF-α and IL-1β by ELISA, NF-κB nuclear translocation by immunofluorescence, and phospho-STAT3 by Western blotting or flow cytometry to clarify whether IL-6 suppression translates into downstream attenuation of pro-survival signaling.

### 3.8. Comparative Analysis of Treatment Effects Across Endpoints

To comprehensively evaluate the relative contributions of treatment formulation and drug concentration to the observed anticancer effects, a comparative analysis of partial eta-squared (η^2^) values across all experimental endpoints was performed (Table 9). This analysis reveals distinct patterns of treatment response depending on the biological endpoint assessed.

For spheroid viability inhibition, treatment formulation (nanoparticle vs. free drug) accounted for 59.1% of the variance, indicating that the delivery system is the dominant determinant of efficacy in the 3D tumor model. In contrast, for clonogenic inhibition, drug concentration was the dominant factor (90.5% of variance), suggesting that long-term reproductive integrity is primarily concentration-dependent, although the nanoparticle formulation still conferred significant advantages at lower concentrations. Most strikingly, for IL-6 suppression, treatment formulation accounted for 83.4% of the variance, indicating that nanoparticle-mediated delivery is the critical factor in modulating inflammatory signaling. These findings collectively demonstrate that SBA-16 nanoparticles enhance the anticancer efficacy of simvastatin through multiple complementary mechanisms, with the relative importance of the delivery system varying according to the specific biological endpoint.

## 4. Discussion

The present study provides comprehensive evidence that encapsulation of simvastatin within SBA-16 mesoporous silica nanoparticles substantially enhances its anticancer efficacy against HT-29 colorectal cancer cells in both two-dimensional monolayer and three-dimensional spheroid culture models. The key findings of this investigation include: (1) successful synthesis and characterization of SBA-16 nanoparticles with favorable physicochemical properties for drug delivery (high surface area, uniform pore size, and appropriate particle size); (2) high drug-loading efficiency (73.83%) and markedly accelerated release kinetics for simvastatin-loaded SBA-16 NPs compared to free drug; (3) a 6.29-fold reduction in IC_50_ (from 27.86 to 4.43 µg/mL) in 2D culture, indicating substantially enhanced cytotoxic potency; (4) superior inhibition of colony formation and spheroid viability by Sim-SBA-16 NPs, particularly at lower concentrations; and (5) marked suppression of IL-6 secretion by the nanoparticle formulation [11,16,39], suggesting modulation of inflammatory pathways implicated in tumor progression. Collectively, these findings establish SBA-16 nanoparticles as a promising drug delivery platform for augmenting the therapeutic efficacy of simvastatin in colorectal cancer [29,40,41].

The 6.29-fold enhancement in cytotoxic potency observed with Sim-SBA-16 NPs compared to free simvastatin is a notable in vitro finding that may support future translational development, provided that selectivity, safety, and in vivo efficacy are confirmed. This magnitude of potency enhancement is consistent with previous reports of mesoporous silica nanoparticle-mediated delivery of other hydrophobic anticancer agents. The superior performance of SBA-16 nanoparticles in the present study can be attributed principally to their high surface area, pore volume, and three-dimensional cage-like pore structure, which enable efficient drug loading and rapid liberation of dissolved simvastatin under the sink conditions tested. Because the present release data show near-complete release within 30 min, the system should not be described as providing sustained release without additional longer-term and physiologically relevant release studies.

The high encapsulation efficiency of 73.83% deserves particular mechanistic consideration in the context of the drug’s physicochemical profile and the unique structural attributes of SBA-16. The loading mechanism is best understood as a multi-step, multi-force process. During solvent impregnation in ethanol, simvastatin—which is highly soluble in ethanol but poorly soluble in water (~0.03 mg/mL)—partitions preferentially into the mesoporous interior driven by capillary condensation, a phenomenon in which the curved meniscus inside nanoscale pores lowers the chemical potential of the solute relative to the bulk solution. The three-dimensional interconnected Im3m cage structure of SBA-16 is particularly advantageous in this regard: unlike the one-dimensional cylindrical channels of MCM-41 or the parallel hexagonal channels of SBA-15, the SBA-16 cage network provides access to the pore interior from multiple directions, minimizing diffusion path lengths and facilitating uniform drug distribution throughout the particle. Upon solvent removal by rotary evaporation, the evaporating ethanol front recedes progressively into the pore interior, depositing simvastatin as a thin amorphous film on the pore walls rather than as a bulk crystalline precipitate—a process that maximizes the drug-surface contact area. The resulting amorphous state of mesopore-confined simvastatin, confirmed by the loss of crystalline XRD peaks (Figure 3C(, represents a thermodynamically metastable but kinetically trapped configuration that is essential for high encapsulation efficiency. In this state, simvastatin lacks the lattice energy of its crystalline form, rendering it more susceptible to dissolution and improving its thermodynamic activity in aqueous media. The surface chemistry of calcined SBA-16 further reinforces drug retention: the high density of surface silanol groups (estimated at ~3–4 OH/nm^2^ for calcined silica) forms hydrogen bonds with the C-13 hydroxyl group and the lactone carbonyl of simvastatin, as evidenced by the red-shift and broadening of the Si–OH stretching band (~960 cm^−1^) and the attenuation of the simvastatin carbonyl peak (~1730 cm^−1^) in the FTIR spectrum of Sim-SBA-16 NPs (Figure 5C). The hydrophobic methyl substituents and the decalin ring system of simvastatin additionally contribute through van der Waals interactions with partially dehydroxylated silica surface patches, providing a secondary retention force that supplements hydrogen bonding. Collectively, this mechanistic framework—capillary condensation driving entry, amorphous confinement stabilizing the loaded state, and synergistic hydrogen bonding and hydrophobic interactions anchoring the drug to the pore walls—provides a coherent and comprehensive explanation for the high EE of 73.83% and underscores the structural suitability of SBA-16 as a nanocarrier for lipophilic drug candidates such as simvastatin [34,42,43].

The markedly accelerated release kinetics observed with Sim-SBA-16 NPs (91.93% cumulative release at 30 min) compared to free simvastatin (35.24%) address a critical pharmacotechnical limitation of this drug. Simvastatin exhibits extremely poor aqueous solubility, which restricts dissolution and bioavailability. The improved release from SBA-16 nanoparticles can be explained by increased effective surface area, enhanced wetting of the hydrophilic silica framework, amorphous confinement of the drug within the mesopores, and multiple diffusion pathways through the interconnected cage-like structure. Importantly, this release pattern should be interpreted as rapid and near-complete liberation under Tween 80-containing sink conditions rather than sustained release. Such rapid release may be advantageous for achieving high dissolved drug availability in vitro, but it may also be detrimental for prolonged systemic therapy if extracellular release occurs before tumor accumulation. Therefore, future studies should evaluate pH-dependent release, serum/plasma stability, protein corona effects, and surface-engineering strategies designed to improve extracellular stability while preserving intracellular drug availability.

A limitation of the present study is that drug release was evaluated only at physiological pH (7.4). Additionally, release studies under physiologically relevant and tumor-mimicking conditions would provide a more comprehensive assessment of nanoparticle performance. In particular, future investigations should examine release behavior under acidic tumor microenvironment conditions (pH~6.5), endo-lysosomal conditions following cellular internalization (pH~5.0), and protein-rich media such as PBS supplemented with fetal bovine serum (FBS) to evaluate the influence of protein corona formation on release kinetics. Furthermore, extending the release study beyond the current observation period would help determine whether residual drug remains associated with the carrier and whether secondary release phases occur over longer timescales. Consequently, although the current release data demonstrate the ability of SBA-16 nanoparticles to rapidly enhance simvastatin dissolution, additional studies under biologically relevant conditions are necessary before definitive conclusions regarding in vivo release behavior and therapeutic performance can be established.

The superior efficacy of Sim-SBA-16 NPs in the clonogenic assay is particularly noteworthy, as this assay provides a stringent measure of long-term cytotoxicity and reproductive integrity. The ability of Sim-SBA-16 NPs to achieve 93.9 ± 3.0% inhibition of colony formation at 30 µg/mL, compared with 14.0 ± 2.5% for free simvastatin, indicates that nanoparticle formulation substantially reduces the clonogenic potential of HT-29 cells. This finding is relevant because residual cancer cells with retained proliferative capacity are a major contributor to recurrence and therapeutic failure [44]. However, the present clonogenic analysis is limited to colony number inhibition and does not include colony size distribution or drug-washout/re-seeding studies. Therefore, the data demonstrate reduced clonogenic survival, but cannot distinguish fully between irreversible cytotoxicity and prolonged cytostatic suppression among surviving cells. Future studies should quantify colony area/size distribution using digital image analysis and include washout-recovery experiments to determine whether Sim-SBA-16 NPs induce durable proliferative arrest or irreversible cell death.

The three-dimensional spheroid model employed in this study provides a more physiologically relevant platform for evaluating anticancer efficacy than conventional 2D monolayer cultures. Spheroids recapitulate key features of solid tumors, including three-dimensional architecture, cell–cell and cell–matrix interactions, hypoxic gradients, and diffusion barriers that limit drug penetration [36]. The superior efficacy of Sim-SBA-16 NPs in the spheroid model supports the functional advantage of the nanoparticle formulation in a 3D context. However, the present data do not directly demonstrate nanoparticle penetration depth or spatial distribution within spheroids. Therefore, statements regarding spheroid penetration should be interpreted cautiously. Future experiments should use fluorescently labelled SBA-16 nanoparticles with confocal z-stack imaging, quantitative radial fluorescence profiling, and histological assessment of spheroid architecture to determine whether the nanoparticles penetrate beyond the peripheral proliferative zone and how treatment affects spheroid integrity.

The marked suppression of IL-6 secretion by Sim-SBA-16 NPs is biologically relevant because IL-6 contributes to CRC progression through JAK/STAT3-associated survival [16,39], proliferation, angiogenesis, and therapy-resistance pathways [45,46,47,48,49,50]. Clinically, elevated circulating IL-6 levels have been repeatedly associated with advanced disease stage, poor prognosis, and reduced survival in colorectal cancer patients, underscoring the translational relevance of IL-6 suppression as a therapeutic endpoint [51,52,53,54,55]. reduction observed with Sim-SBA-16 NPs than with free simvastatin suggests that nanoparticle formulation may enhance the anti-inflammatory activity of simvastatin. Nevertheless, the present study measured secreted IL-6 only; it did not assess JAK/STAT3 activation, NF-κB signaling, TNF-α, IL-1β, or apoptosis-linked downstream targets [56,57,58,59,60,61,62]. Therefore, IL-6 suppression should be interpreted as a supportive mechanistic marker rather than proof of a complete inflammatory signaling mechanism. Future experiments should directly test phospho-STAT3, NF-κB nuclear translocation, TNF-α/IL-1β secretion, and apoptosis markers to establish whether IL-6 suppression causally contributes to the enhanced cytotoxic and spheroid-inhibitory effects [56,57,58,59,60,61,62].

The mechanisms underlying the anticancer effects of simvastatin have been extensively investigated and involve multiple complementary pathways. As a competitive inhibitor of HMG-CoA reductase, simvastatin blocks the mevalonate pathway and reduces downstream isoprenoid intermediates such as farnesyl pyrophosphate and geranylgeranyl pyrophosphate, which are required for prenylation of Ras, Rho, and Rac GTPases [63,64,65,66,67,68]. Disruption of these pathways can impair oncogenic signaling, proliferation, survival, migration, and invasion [69]. Statins have also been reported to modulate inflammatory signaling pathways, including NF-κB and STAT3 [70]. In the present study, these known mechanisms provide biological context for the observed cytotoxic and IL-6-suppressive effects; however, direct measurements of apoptosis, necrosis, mitochondrial dysfunction, cell-cycle arrest, Ras/Rho prenylation, or NF-κB/STAT3 activity were not performed. Therefore, the mechanistic interpretation should remain cautious. Future studies should include Annexin V/PI staining, caspase-3/7 activity, Bcl-2/Bax/PARP analyses, mitochondrial membrane potential assessment, and cell-cycle profiling to define the mode of cell death and the signaling pathways affected by Sim-SBA-16 NPs.

The physicochemical properties of SBA-16 nanoparticles synthesized in this study are favorable for drug delivery applications. The mean hydrodynamic diameter of 31.67 nm falls within a sub-100 nm range commonly associated with efficient cellular interaction and possible endocytic internalisation, although direct uptake was not measured in this study. The moderately negative zeta potential (−18.5 mV) provides electrostatic repulsion that supports colloidal stability. The high BET surface area (742 m^2^/g) and large pore volume (0.58 cm^3^/g) enable substantial drug loading, while the uniform pore size (6.8 nm) is well suited for simvastatin loading. These physicochemical properties likely contribute to the improved in vitro performance of SBA-16 nanoparticles as a delivery platform for simvastatin.

A critical methodological question raised by the reviewer’s comment concerns whether the SBA-16 nanoparticle carrier itself contributes to the observed biological effects—cytotoxicity, clonogenic inhibition, spheroid suppression, and IL-6 reduction—independently of simvastatin loading. This question has fundamental implications for the mechanistic attribution of the observed effects: if blank SBA-16 NPs exert significant biological activity at the concentrations tested, then a portion of the efficacy attributed to “Sim-SBA-16 NPs” may in fact reflect carrier-mediated effects rather than simvastatin-specific pharmacology, confounding the interpretation of the 6.29-fold IC_50_ reduction and the IL-6 suppression data. The present study did not include a blank SBA-16 NP control arm, which is acknowledged as a limitation. The following discussion evaluates the likelihood and nature of carrier-independent biological effects based on the established literature on mesoporous silica nanoparticle biocompatibility, and proposes the experimental framework for resolving this question. Regarding intrinsic cytotoxicity of SBA-16 nanoparticles: the biocompatibility of calcined mesoporous silica nanoparticles in mammalian cell lines has been extensively studied, and the broad consensus of the literature is that bare amorphous silica nanoparticles exhibit relatively low cytotoxicity in the 0–100 µg/mL concentration range in most cancer cell lines, with cell viability typically remaining above 80–90% at concentrations below 50 µg/mL after 24–48 h of exposure [43,71]. This biocompatibility arises from the chemical inertness of the amorphous silica framework, the absence of redox-active metal species, and the high hydrophilicity of the silanol-rich surface that minimizes membrane disruption. Specifically for SBA-16, the three-dimensional cage-like pore structure with large pore windows (5–10 nm) has been shown to promote serum protein adsorption (forming a protein corona) that further reduces non-specific membrane interaction and attenuates cytotoxicity relative to smaller pore materials [43,72]. However, it is important to note that cytotoxicity of mesoporous silica nanoparticles is dose-dependent, particle-size-dependent, and surface-chemistry-dependent [28], and that at the high end of the concentration range tested in this study (60 µg/mL), some intrinsic cytotoxicity cannot be excluded a priori. Furthermore, the surface silanol density of calcined SBA-16 is high (~3–4 SiOH/nm^2^), and reactive surface silanols have been shown to generate low-level reactive oxygen species (ROS) upon cellular internalization through their interaction with reducing agents in the lysosomal environment, a mechanism that can contribute to mitochondrial stress and apoptosis at high nanoparticle concentrations [71]. For the IL-6 endpoint specifically, there is a literature precedent suggesting that mesoporous silica nanoparticles can modulate inflammatory cytokine production independently of drug loading: silica particles have been shown to activate the NLRP3 inflammasome and stimulate IL-1β production in macrophages and dendritic cells through surface silanol–membrane interactions [73], although this effect is more pronounced for larger crystalline silica particles (>2 µm) than for the amorphous nanoparticle SBA-16 used here. More relevantly, several studies have shown that mesoporous silica nanoparticles at sub-cytotoxic concentrations can suppress NF-κB activation and reduce IL-6 secretion in epithelial cells through a mechanism involving silanol-mediated inhibition of IKKβ activity—an effect that is independent of any loaded drug and that would confound the interpretation of the IL-6 data if blank SBA-16 NPs exert this effect at 15 and 30 µg/mL. The magnitude and direction of this carrier-mediated IL-6 modulation are uncertain for SBA-16 specifically in HT-29 cells, and can only be resolved by including blank SBA-16 NP controls in the ELISA. To definitively address the reviewer’s concern, the following experimental approach is proposed as a high-priority addition to future studies: (i) MTT viability assay with blank SBA-16 NPs at matched mass concentrations (5–60 µg/mL) to establish whether the carrier is cytotoxic at the doses used in this study; (ii) IL-6 ELISA with blank SBA-16 NPs at 15 and 30 µg/mL to determine whether the carrier independently suppresses IL-6 secretion; (iii) clonogenic assay with blank SBA-16 NPs at 15, 30, and 60 µg/mL to establish whether blank carrier independently inhibits colony formation; and (iv) 3D spheroid viability assay with blank SBA-16 NPs at matched concentrations to confirm that spheroid disintegration is drug-dependent. The critical analytical output from this experimental set would be: if blank SBA-16 NPs produce cell viability ≥90%, IL-6 levels not significantly different from untreated controls, and colony formation efficiency ≥90% of untreated controls across the tested concentration range, then all observed biological effects of Sim-SBA-16 NPs can be confidently attributed to the delivered simvastatin, validating the mechanistic interpretations presented in this manuscript. Conversely, any significant carrier-independent activity would require a revised attribution of the observed effects and subtraction of the carrier contribution from the total measured efficacy of Sim-SBA-16 NPs, potentially modifying the claimed 6.29-fold IC_50_ reduction and the IL-6 suppression data. Based on the available literature on SBA-16 biocompatibility and the sub-cytotoxic concentrations at which the critical IC_50_ difference between free simvastatin and Sim-SBA-16 NPs is observed (4.43 vs. 27.86 µg/mL), it is mechanistically unlikely that carrier-independent cytotoxicity accounts for the majority of the observed effect; however, formal experimental confirmation through a blank SBA-16 control arm is essential for rigorous mechanistic attribution and is explicitly recommended for the next iteration of this study.

The 6.29-fold reduction in IC_50_ observed for Sim-SBA-16 NPs relative to free simvastatin is most conservatively explained by improved drug solubilisation, amorphous confinement, and rapid release of dissolved simvastatin from the SBA-16 carrier. Additional mechanisms, including nanoparticle-cell interaction, possible endocytic internalisation, intracellular drug accumulation, and altered intracellular trafficking, may also contribute; however, these were not directly demonstrated in the present study. Therefore, the uptake-related mechanism should be regarded as a plausible hypothesis rather than established evidence. Direct confirmation will require fluorescently labelled nanoparticles, quantitative uptake studies, intracellular simvastatin measurement, endocytosis-inhibition experiments, and subcellular localization analysis. This cautious interpretation avoids over-attribution while preserving the biological rationale for the enhanced functional efficacy observed in the MTT, clonogenic, spheroid, and IL-6 assays.

Comparison of the present findings with previous studies on statin-based anticancer therapies reveals both consistencies and novel contributions. Several preclinical studies have reported anticancer effects of statins in colorectal cancer models, with IC_50_ values for free simvastatin typically ranging from 20 to 50 µg/mL in 2D culture, consistent with the 27.86 µg/mL observed in the present study [74,75]. However, few studies have systematically evaluated nanoparticle-mediated delivery of statins in CRC models, and to our knowledge, this is the first report demonstrating the superior efficacy of simvastatin-loaded SBA-16 nanoparticles in both 2D and 3D HT-29 cell culture systems. Previous investigations of mesoporous silica nanoparticle delivery systems have primarily focused on conventional chemotherapeutics such as doxorubicin, paclitaxel, and cisplatin [40,76], with limited attention to repurposed drugs such as statins [22]. The present study extends this body of work by demonstrating that nanoparticle-mediated delivery can substantially enhance the anticancer efficacy of simvastatin, a widely prescribed and well-tolerated drug with established safety profiles in clinical use.

The integration of multiple complementary assays in this study—including MTT viability assay, clonogenic assay, 3D spheroid culture, and IL-6 quantification—provides a broad assessment of anticancer efficacy across different biological endpoints and experimental paradigms. The comparative analysis of effect sizes (Table 9) suggests that the relative importance of nanoparticle-mediated delivery varies according to the endpoint assessed. These findings support the conclusion that SBA-16 nanoparticles enhance the in vitro anticancer efficacy of simvastatin mainly through improved formulation performance, including drug loading, solubilisation, and rapid release. Potential contributions from enhanced uptake, intracellular accumulation, and spheroid penetration remain mechanistic hypotheses that require direct experimental validation.

Despite the promising findings of this study, several limitations must be acknowledged. First, all experiments were conducted in vitro using a single colorectal cancer cell line (HT-29), and future studies should evaluate additional CRC cell lines with diverse genetic backgrounds. Second, no in vivo pharmacokinetic, biodistribution, efficacy, or toxicity studies were performed. Third, cellular uptake, intracellular trafficking, and intracellular simvastatin accumulation were not directly measured, so endocytic internalisation remains a hypothesis. Fourth, the study did not include blank SBA-16 nanoparticle controls, preventing formal separation of carrier-related effects from simvastatin-specific effects. Fifth, cytotoxicity was not assessed in normal colonic epithelial cells; therefore, selectivity index and therapeutic window cannot yet be established. Sixth, MTT data were not complemented by apoptosis, necrosis, mitochondrial dysfunction, or cell-cycle assays. Seventh, IL-6 was the only inflammatory mediator measured, and TNF-α, IL-1β, NF-κB, and STAT3 signaling require evaluation. Finally, the release study was performed only in PBS pH 7.4 with 0.5% Tween 80 over a short time window. Future studies should include tumor-mimicking acidic pH, endo-lysosomal pH, albumin/serum/plasma-containing media, spheroid penetration studies, and in vivo models to define translational potential more rigorously.

The translational potential of simvastatin-loaded SBA-16 nanoparticles for colorectal cancer therapy is supported by several considerations. First, simvastatin is a widely prescribed drug with well-established safety profiles and decades of clinical use, which may facilitate regulatory approval and clinical translation. Second, the pleiotropic anticancer mechanisms of statins—including inhibition of oncogenic signaling, induction of apoptosis, suppression of angiogenesis, and modulation of inflammatory pathways—provide multiple complementary avenues for therapeutic intervention. Third, the dual mechanism of action of Sim-SBA-16 NPs, encompassing both direct cytotoxic effects and anti-inflammatory activity, may be particularly advantageous in the context of CRC, where chronic inflammation plays a central role in tumor initiation and progression. Fourth, the potential for combination therapy with conventional chemotherapeutics or targeted agents represents an attractive strategy for enhancing therapeutic efficacy while minimizing toxicity. Statins have been shown to synergize with various anticancer agents, including 5-fluorouracil, oxaliplatin, and cetuximab, in preclinical CRC models [74,75,76,77,78], and nanoparticle-mediated co-delivery of simvastatin with other drugs could further improve therapeutic outcomes [40,79,80].

## 5. Conclusions

This study demonstrates that encapsulation of simvastatin within SBA-16 mesoporous silica nanoparticles substantially enhances its in vitro anticancer efficacy against HT-29 colorectal cancer cells. The synthesized SBA-16 nanoparticles exhibited favorable physicochemical properties, including high surface area (742 m^2^/g), uniform pore size (6.8 nm), appropriate particle size (31.67 nm), and high drug-loading efficiency (73.83%). Simvastatin-loaded SBA-16 nanoparticles demonstrated rapid and near-complete release under sink conditions (91.93% cumulative release at 30 min) compared to free drug (35.24%), addressing the limitation of poor aqueous solubility. In two-dimensional monolayer culture, Sim-SBA-16 NPs achieved a 6.29-fold reduction in IC_50_ (from 27.86 to 4.43 µg/mL, *p* < 0.001). The nanoparticle formulation also exhibited superior efficacy in clonogenic and three-dimensional spheroid assays and induced marked suppression of IL-6 secretion (72.2% reduction at 30 µg/mL), suggesting potential modulation of inflammatory signaling pathways implicated in tumor progression.

Overall, the findings support SBA-16 nanoparticles as a promising formulation strategy for improving the in vitro performance of simvastatin in colorectal cancer models. However, the clinical implications remain preliminary because direct uptake data, blank-carrier controls, normal-cell selectivity, detailed cell-death mechanisms, broader inflammatory profiling, physiologically relevant release studies, and in vivo validation are still required. These additional studies will be essential before drawing firm conclusions about therapeutic dosing, safety, or clinical translation.

Future investigations should focus on: (1) comprehensive in vivo pharmacokinetic studies to evaluate biodistribution, tumor accumulation, and systemic clearance of Sim-SBA-16 NPs in preclinical CRC models; (2) efficacy studies in orthotopic and patient-derived xenograft models; (3) mechanistic studies using labelled nanoparticles, endocytosis inhibitors, and intracellular drug quantification to elucidate uptake and trafficking; (4) testing across a panel of CRC cell lines and normal colonic epithelial cells to assess generalizability and selectivity; (5) inclusion of blank SBA-16 controls to separate carrier effects from drug effects; (6) apoptosis, mitochondrial dysfunction, cell-cycle, and broader inflammatory pathway analyses; and (7) release studies in tumor-mimicking, endo-lysosomal, albumin-containing, serum-containing, and plasma-containing media. Collectively, these directions will provide a stronger foundation for advancing simvastatin-loaded SBA-16 nanoparticles toward translational evaluation.

## Figures and Tables

**Table 1:** Cumulative release of Simvastatin from SBA-16 nanoparticles and free drug.

Time (min)	SBA-16 NPs (%)	Free Drug (%)
0	0.00	0.00
5	38.20	5.10
10	58.60	9.30
15	72.40	13.80
20	82.10	18.20
25	88.50	21.90
30	91.93	35.24

**Table 2:** IC_50_ values and fold change in cytotoxic potency.

Formulation	IC_50_ (µg/mL)	Fold Change	Significance
Free Simvastatin	27.86	1.00× (reference)	—
Simvastatin/SBA-16 NPs	4.43	6.29× more potent	*p* < 0.001 (***)

*** corresponds to *p* < 0.001 compared with the free simvastatin group.

**Table 3:** Percentage inhibition of colony formation.

Formulation	15 µg/mL	30 µg/mL	60 µg/mL	Trend
Free Simvastatin	3.0 ± 1.5	14.0 ± 2.5	44.0 ± 4.0	Moderate increase
Simvastatin/SBA-16 NPs	37.0 ± 3.5	93.9 ± 3.0	96.0 ± 2.0	Rapid plateau

**Table 4:** Two-way ANOVA for colony formation inhibition.

Source of Variation	SS	Df	MS	F	*p*-Value	Sig.	η^2^
Treatment (Formulation)	13,761.405	1	13,761.405	1936.337	<0.0001	***	0.5913
Concentration (Dose)	7820.410	2	3910.205	550.208	<0.0001	***	0.3360
Treatment × Concentration	1604.610	2	802.305	112.893	<0.0001	***	0.0690
Within (Error)	85.281	12	7.107	—	—	—	—
Total	23,271.706	17	—	—	—	—	—

*** indicates statistical significance at *p* < 0.0001.

**Table 5:** Percentage inhibition of spheroid viability.

Formulation	15 µg/mL	30 µg/mL	60 µg/mL	Concentration-Response Trend
Free Simvastatin	3.0 ± 1.5	14.0 ± 2.5	44.0 ± 4.0	Gradual, sub-maximal increase
Simvastatin/SBA-16 NPs	37.0 ± 3.5	93.9 ± 3.0	96.0 ± 2.0	Steep increase; plateau at 30 µg/mL

**Table 6:** Spheroid morphometric data: equivalent circular diameter (px and µm), cross-sectional area, estimated volume, and SGI% for all treatment groups. Diameter was calculated as D = 2 × (Area/π)^1/2^ from ImageJ (Version 1.54, National Institutes of Health, Bethesda, MD, USA)-measured cross-sectional area; volume was estimated as V = πD^3^/6. Values are presented as mean ± SD where applicable.

Group	Conc.	*n*	Diameter (µm)	Diameter (µm)	Area (px^3^)	Vol. (×10^9^ px^3^)	SGI (%)
Untreated control	-	1	1117	558.7	980,643	0.73	-
SBA-16 carrier control	-	2	1258 ± 188	629.1 ± 94	1,257,451	1.08	-
Free SIM	15 µg/mL	2	1279 ± 47	639.6 ± 23	1,286,063	1.10	3.0
Free SIM	30 µg/mL	2	1170 ± 110	584.9 ± 55	1,079,469	0.85	14.0
Free SIM	60 µg/mL	1	1311	655.6	1,350,139	1.18	44.0
SBA-16/SIM	15 µg/mL	2	1003 ± 49	501.7 ± 25	791,725	0.53	37.0
SBA-16/SIM	30 µg/mL	2	240 ± 101	120.2 ± 50	49,340	0.01	93.9
SBA-16/SIM	60 µg/mL	2	255 ± 41	127.3 ± 21		0.01	96.0

**Table 7:** IL-6 secretion levels (pg/mL) following treatment.

Formulation	15 µg/mL	30 µg/mL	Fold Reduction (30 µg/mL)
Untreated Control	245.3 ± 18.7	245.3 ± 18.7	—
Free Simvastatin	198.4 ± 15.2	156.7 ± 12.4	1.56-fold
Simvastatin/SBA-16 NPs	142.6 ± 10.8	68.3 ± 8.6	3.59-fold

**Table 8:** Two-way ANOVA for IL-6 suppression.

Source of Variation	SS	Df	MS	F	*p*-Value	Sig.	η^2^
Treatment (Formulation)	13,739.717	1	13,739.717	1079.171	<0.0001	***	0.8337
Concentration (Dose)	1753.292	1	1753.292	137.710	<0.0001	***	0.1064
Treatment × Concentration	884.942	1	884.942	69.507	<0.0001	***	0.0537
Within (Error)	101.854	8	12.732	—	—	—	—
Total	16,479.804	11	—	—	—	—	—

*** indicates statistical significance at *p* < 0.0001.

**Table 9:** Comparative effect sizes across experimental endpoints.

Endpoint	η^2^ Treatment	Treatment Sig.	η^2^ Concentration	η^2^ Interaction	Dominant Factor
Spheroid Inhibition	0.5913	***	0.3360	0.0690	Treatment (59.1%)
Clonogenic Inhibition	0.0181	*	0.9051	0.0362	Concentration (90.5%)
IL-6 Inhibition	0.8337	***	0.1064	0.0537	Treatment (83.4%)

* indicates statistical significance at *p* < 0.05; *** indicates statistical significance at *p* < 0.0001.

## Data Availability

The data presented in this study are available from the corresponding author upon reasonable request.

## Abbreviations

The following abbreviations are used in this manuscript:

2D	Two-Dimensional
3D	Three-dimensional
ANOVA	Analysis of Variance
APC	Adenomatous Polyposis Coli
ATCC	American Type Culture Collection
BET	Brunauer–Emmett–Teller
BJH	Barrett-Joyner-Halenda
BRAF	B-Raf Proto-Oncogen
CO_2_	Carbon dioxide
CRC	Colorectal Cancer
DLC	Drug Loading Content
DLS	Dynamic Light Scattering
DMEM	Dulbecco’s Modified Eagle Medium
DMSO	Dimethyl Sulfoxide
EE	Encapsulation Efficiency
ELISA	Enzyme-Linked Immunoassay Assay
F127	Pluronic F127
FBS	Fetal Bovine Serum
FPP	Farnesyl Pyrophosphate
FTIR	Fourier-Transform Infrared Spectroscopy
GGPP	Geranylgeranyl Pyrophosphate
HCl	Hydrochloric Acid
HMG-CoA	3-Hydroxy-3-Methylglutaryl-Coenzyme A
HPLC	High-Performance Liquid Chromatography
HT-29	Human Colorectal Adenocarcinoma Cell line
IC_50_	Half-Maximum Inhibitory Concentration
IL-6	Interleukin-6
SBA-16	Santa Barbara Amorphous-16
SD	Standard Deviation
SEM	Scanning Electron Microscopy
SIM-SBA-16	Simvastatin-Loaded SBA-16
NPs	Nanoparticles
STAT3	Signal Transducer and Activator of Transcription 3
TEOS	Tetraethyl Orthosilicate
TEM	Transition Electron Microscopy
TME	Tumor Microenvironment
TP53	Tumor Protein p53
UV-Vis	Ultraviolet-Visible Spectrophotometry
JAK	Janus Kinase
KRAS	Kirsten Rat Sarcoma Viral Oncogene
MCM-41	Mobile Composition of Matter No. 41
MS	Mass Spectrometry
MSNs	Mesoporous Silica Nanoparticles
MTT	3-(4,5-Dimethylthiazole-2-yl)-2,5-Diphenyltetrazolium Bromide
NF-kB	Nuclear Factor Kappa B
PBS	Phosphate-Buffered Saline
PDI	Polydispersity Index
